# Manipulation of the polarization of Terahertz wave in subwavelength regime

**DOI:** 10.1038/srep08306

**Published:** 2015-02-06

**Authors:** Xiao Xiao, Ho Ming Leung, C. T. Chan, Weijia Wen

**Affiliations:** 1 Department of Physics, Hong Kong University of Science and Technology Clear Water Bay, Kowloon, Hong Kong, China

## Abstract

By generalizing the concept of spoof surface Plasmons (*Science* 305, 847), we analytically demonstrate that subwavelength quarter-wave and half-wave plates can be realized in a metal hole array (MHA) sandwiched by two thin-layer materials, whose optical responses can be characterized by their optical conductivities. These abilities of polarization conversion can be attributed to the novel eigenstates induced by the hybridization of the spoof surface plamsons with the current generated in the thin-layer. Due to this mechanism, the robustness of the system is promised. The analytic predictions are verified numerically by modeling the thin-layer material as an experimentally feasible topological-insulator/SiO_2_ multilayer. Moreover, the possibility of extending the principle to a broad range of materials is dicussed.

Manipulating the polarization of a terahertz (THz) wave, especially that of the circularly polarized THz wave, has proved to be challenging^1^, because most THz sources are based on optical rectification^2^ or transient dipole radiation^3^. Typically, the manipulation of the polarization of THz wave can be achieved by using THz polarization pulse shaping technology^4^, fabricating helix electrodes^5^, building a Fresnel prism^6^, or using birefringent metamaterial^7^. However, helix electrodes can only generates elliptically polarized light, and the later two approaches (Fresnel prism and birefringent metamaterial) introduce substantial material loss and Fresnel loss, because of their thicknesses comparable with wavelength. Thus, the working bandwidth and the intensity of THz wave are rather limited. On the other hand, the existing proposals of wave plate, which base on the accidental fit of the polarization conversion conditions, have either low conversion efficiency^8^,^9^,^10^,^11^,^12^ or special requirements to the incident direction and polarization^13^. To overcome these limits, we will explore in this work some possible eigenstates, which can serve as THz quarter-wave and half-wave plates working in the subwavelength regime.

Let us consider a metal hole array (MHA)^14^,^15^,^16^ sandwiched by two thin-layer materials supporting in-layer currents. The thickness of each thin-layer is much smaller than the wavelength of interest so that the response of the layers to the external electromagnetic field can be described by the optical conductivities of the layers. We suppose that the conductivities in the two layers are the same in magnitudes and denoted as *σ_xx_* (the longitudinal conductivity) and *σ_xy_* (the Hall conductivity). Further, we denote the periodicity of the MHA as *d*. Throughout the study, the side length of the square holes *l* and the thickness of the MHA *h* are set to be *l* = *d/3* and *h* = *d/10* respectively. Without loss of the generality the normally incident *x*-polarized light is assumed to propagate upward along *z*-direction. By generalizing the mode expansion formalism^16^,^17^,^18^, a group of equations coupling the modes at the two sides of MHA are obtained^19^ with mathematical details outlined in the method section:
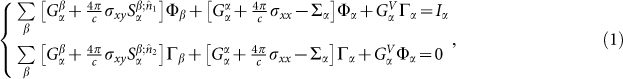
where Φ*_α_* and Γ*_α_* denote the fields of *α* waveguide mode at the incident and transmitted sides of MHA respectively, 
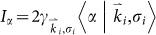
 indicates the coupling of the incident field and the α waveguide mode, 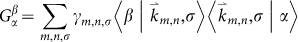 gives the coupling between different waveguide modes at the given side of MHA, 
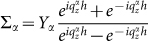
 is due to the bouncing back and forth of the *α* waveguide mode in holes, 
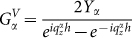
 measures the coupling strength between the *α* waveguide mode at the two sides of holes, and 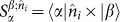 is the coupling between different waveguide modes in the presence of optical Hall currents in the lower (*i* = 1) or upper (*i* = 2) thin layer. In the above expressions,*γ_m,n,σ_* and *Y_α_* are the admittances of the grating modes and waveguide modes,|*α*〉 denotes the tangential component (parallel to the metal surface) of the *α* waveguide mode, 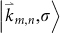 indicates the tangential component of grating modes in open space, the Bra-notation indicates the complex conjugate of the corresponding modes, 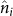 is an unit vector, which together with the electric field determines the direction of Hall current (
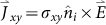
) in the lower or upper thin-layer, and the inner products in the expressions are obtained by integrating the product over the unit cell.

## Results

### Subwavelength quarter and half wave plate

Given that holes are subwavelength, the waveguide modes exponentially decay along the propagating direction. It is a good approximation to consider only the fundamental modes, *i.e.* the transversal electric (TE) modes with indexes (0,1) and (1,0). In the notation (m,n), the indexes m and n are for *x*- and *y*-directions respectively. Taking the vector 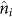 to be along the same direction for the two thin layers, the transmission can be obtained by solving Eq. (1) with the fundamental-mode approximation:
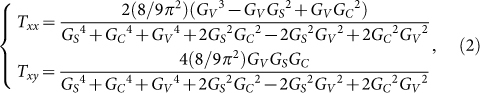
where 
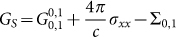
, 
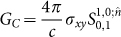
, and 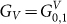. In the above, we have taken the facts: 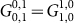, 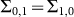, 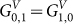 and 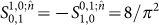 due to the geometry of the holes. As the holes are subwavelength, Σ_0,1_ and *G_V_* are purely imaginary. Moreover, the real part of 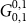 can always be neglected in subwavelength regime. Thus, when the dissipative parts of *σ_xx_* and *σ_xy_* are small, *G_S_* and *G_C_* can be regarded as an imaginary number and a real number respectively. Consequently, the coefficient *T_xx_* would have *π*/4 phase difference from *T_xy_*. Moreover, the two transmission coefficients would be equal to each other in magnitude with 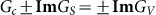. Under the above condition the denominators of the transmission coefficients become vanishing, which means that the resonances of the system coincide with the polarization changes. Following the similar analysis, it can be shown that the reflected light should be also circularly polarized (see supplementary material). Moreover, one can further verify that the reflected and transmitted lights are of the same strength and handedness at the resonance frequencies of the system (see supplementary material). Based on these observations, we may conclude that ***the system supports circularly polarized eigenstates***, which is exotic for a planar system much thinner than the wavelength.

When the vectors 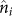 of the two layers point to the opposite directions, the reflection coefficients from the system are given by:
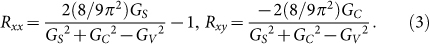
As motioned, when the dissipative parts of *σ_xx_*and *σ_xy_* are small, in subwavelength regime **Im** *G_S_* ≫ **Re** *G_S_* = 8/9*π*^2^, the denominator of the coefficients can be expanded as:
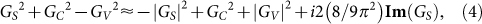
Given the smallness of the imaginary part in the denominator, the resonance of the system is roughly determined by the condition 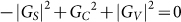. Thus, one observes that at the resonance condition 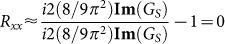. This indicates that the polarization of the reflected light is rotated by 90° at the resonant frequencies of the system. Similar analysis to the transmission coefficients indicates that *T_xy_* simply vanishes (see supplementary material), which means that the polarization of the transmitted light is preserved to be the same with the incident one. According to Eq. (3), the magnitude of the reflected light depends on the magnitude of optical Hall conductivity explicitly.

The phenomena outlined above are the consequence of the splitting of the so-called spoof surface Plasmon^14^ by the surface Hall conductivity (see Eq. (1)). Only when *σ_xy_* is large enough, the resonant modes of the system can be well separated so that the polarization of the outgoing wave is converted completely. The minimal value of *σ_xy_* to achieve complete conversion cannot be obtained analytically and should be determined numerically.

### Numerical verification

**Figure 1:**
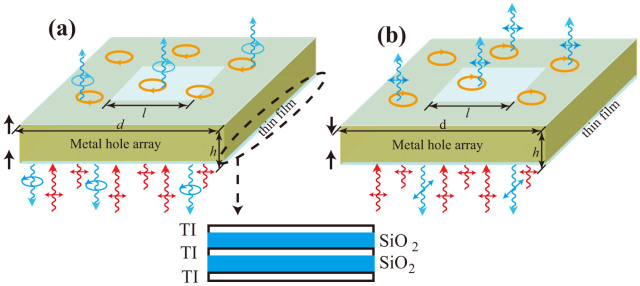
Schematic illustrations of quarter-wave and half-wave plates realized in MHA sandwiched between two thin-layer materials made of TI/SiO_2_ multilayer structure: (a) quarter-wave plate; (b) half-wave plate. The red wavy lines denote the incident linearly polarized wave, blue wavy lines are the transmitted and reflected waves, the circles and arrows in blue denote the polarization of the transmitted and reflected waves, yellow circles denotes the in-layer currents, and the black arrows denote the directions of 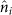 (definitions are in the text) in the lower (*i* = 1) and upper (*i* = 2) thin-layer materials.

To quantitatively verify the above analytical results, we assume that the thin-layer materials have a multilayer structure, consisting of alternating 3D topological insulator (TI) thin film (Bi_2_Se_3_) and dielectric SiO_2_ spacer film (See Fig. 1). As it has been reported, when the distance between two TI surfaces is smaller than 5 quintuple layers (~5 nm), the Dirac fermions on the neighbored TI surfaces would be hybridized and hence gapped^19^. Moreover, the sign of the gap depends on the distance^20^,^21^. We further assume that each TI layer is isolated by SiO_2_ space layers of sufficient thicknesses (*i.e.*>5 nm) so that adjacent TI layers are decoupled. The thickness of a multilayer containing a few tens of TI/SiO_2_ unit cells can still be much smaller than the typical THz wavelength (~300 μm) so that the treatment of the multilayer as thin film is valid. Considering the success of growing ultrathin high-quality films of Bi_2_Se_3_^21^, such a multilayer structure can be fabricated by available technology. For each TI surface, the gapped fermions are described by the below effective Hamiltonian^23^,^24^,^25^,^26^:
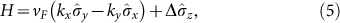
Where *v_F_* is the Fermi velocity, 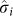 (*i* = *x*, *y*, and *z*) are the Pauli matrices, *k_x_* (*k_y_*) is the x- (y-) component momentum of the quasi-particles, and 2Δ, whose sign determines the direction of 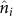, measures the gap between the valence and conduction bands. The optical conductance of the thin-layer containing *N* TI/SiO_2_ unit cells should be 2*N* times of a single TI surface, which can be calculated by Kubo's formula^22^,^23^.

**Figure 2:**
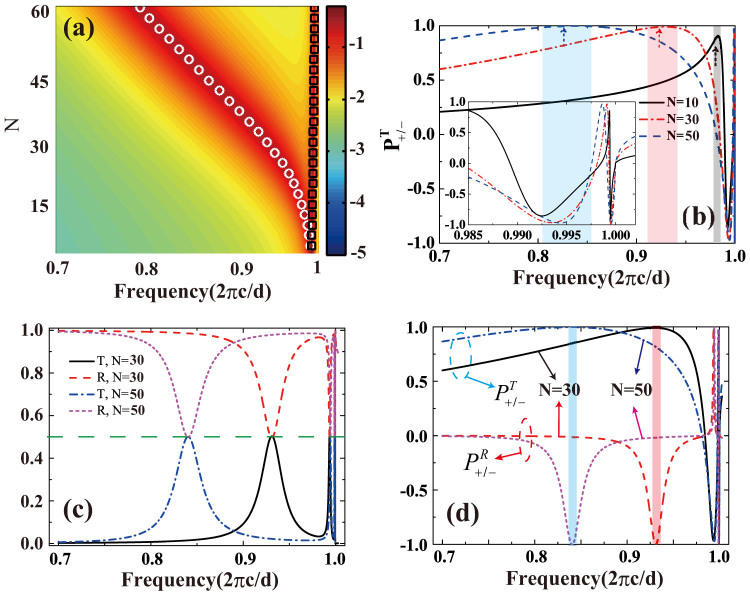
(a) The transmission spectra (log scale) versus number of TI/SiO_2_ unit cells (TI layers are undoped); (b) The quantity 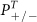 (see text for definition) plotted as a function of frequency for three different TI/SiO_2_ unit cell numbers (*N* = 10, 30, 50): 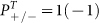 means that the wave is left (right) handed, the three highlighted regions correspond to the nearly circularly polarized regions around the resonant mode denoted by the white dots in (a), and the inset shows the behaviors at around the Wood's anomaly *U/h* = 2*πc/d*. (c) Transmission and reflection spectra for *N* = 30 and *N* = 50: at the resonant frequencies the transmission is equal to the reflection; (d) the quantities 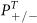 and 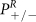 plotted as functions of frequency for *N* = 30 and *N* = 50: at the resonant frequencies (highlighted regions) the handedness of the reflected light and transmitted light are the same. In (a), the white dots and black squares are obtained under the fundamental-mode approximation. In all the calculations, the gap Δ is set to be Δ = 0.6 *U*.

When the energy gaps (2Δ) in the upper and lower multilayers are the same, the quantity 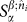 should be of the same sign at the two sides of MHA. The whole system is expected to be a quarter-wave plate. In Fig. 2 (a) we plot the transmission in the log scale as a function of frequency and the number of TI/SiO_2_ unit cells (*N*). Two resonant modes can be identified and they are determined by the conditions **Im** *G_S_* − *G_c_* + **Im** *G_V_* = 0 (the white dot in Fig. 2 (a)) and −**Im** *G_S_* − *G_c_* − **Im** *G_V_* = 0 (the black square in Fig. 2 (a)) respectively. Given the smallness of THz photon energy, the gap can always be made to be larger than the photon energy, so with the increment of *N* the optical Hall conductivity increases. Consequently, the resonant frequencies of the modes become lower, which can be seen clearly from the white dotted curve in Fig. 2 (a). In the meanwhile, to characterize the handedness of the transmitted and reflected light, we define the following quantities:
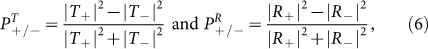
where 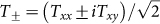 (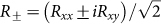) are the transmission (reflection) coefficients for the left (+) and right (−) hand lights respectively. We then plot 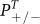 as a function of frequency for *N* = 10, *N* = 30, and *N* = 50 respectively in Fig. 2 (b). It can be seen that the transmitted light near the resonant frequencies are indeed nearly circularly polarized. Moreover, with the increase of the Hall conductivity in layers, the bandwidth of the circularly polarized light becomes wider. In Fig. 2 (c), the transmission and reflection spectra are plotted for *N* = 30 and *N* = 50 cases. As it is expected from previous analytical analysis, the strength of the transmitted light is equal to that of the reflected light at the resonant frequencies. In Fig. 2 (d) both 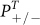 and 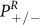 are plotted as functions of frequencies for *N* = 30 and *N* = 50 cases. It proves that at the resonant frequencies the handedness of the transmitted light is the same with reflected light. Based on the model, the minimal number of TI/SiO_2_ unit cells to perform the complete conversion of polarization is about *N* = 20, which means that the minimal *σ_xy_* is about 20 *e*^2^/*h*. Thus, for the *N* = 10 case shown in Fig. 2(b) the value of 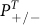 is smaller than 1 accordingly.

**Figure 3:**
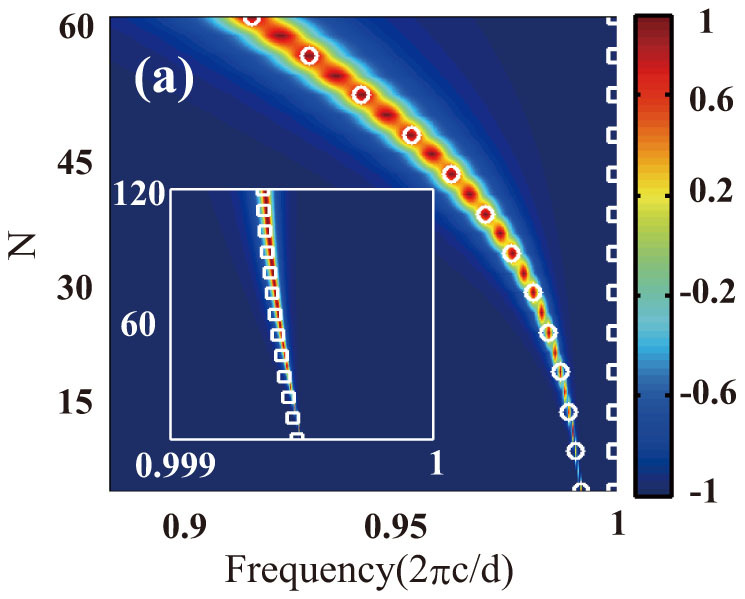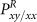 as a function of frequency and number of TI/SiO_2_ unit cells (TI layers are undoped), where 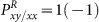 means that the polarization is along *y*- (*x*-) direction. The symbols are the resonant frequencies predicted by following the fundamental-mode approximation.

When the energy gaps in the two multilayers are different in sign, the quantities 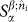 at the two sides of MHA should also be opposite, which means that the system is a half-wave plate. To characterize the polarization of the reflected light, we define the quantity:
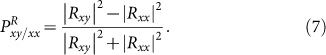
In Fig. 3, we plot 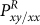 as a function of frequency and TI/SiO_2_ unit cell number *N*. Along the resonant bands (see symbols in Fig. 3), which is determined by the condition 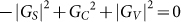, the polarization of the reflected light is indeed rotated by 90° from that of the incident light. One can also see that with the increment of *N*, the resonant frequencies (of both bands) manifest a red-shift and the bandwidth of the polarization rotation is broadened.

## Discussion

When one tunes the gaps of Dirac fermions to be much larger than the photon energy, all the optical conductivities become vanishing, except for the quantized optical Hall conductivity^22^,^23^,^24^,^25^. In this limit, given the accuracy of the fundamental-mode approximation for subwavelength holes, the resonant frequency can relate to the fine structure constant. For examples, in quarter-wave plate case the resonant frequency of the lowest resonant band (white dots in Fig. 2 (a) and (c)) can be related to the fine structure constant α as:
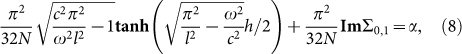
and in half-wave plate case, the resonant frequencies of the resonant bands relates to α like:
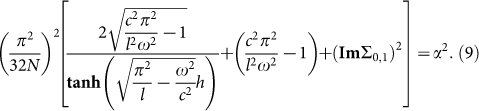
We note that the fine structure constant only relates to the resonant frequency and the geometry parameters of MHA. This is quite different from a pure TI film, where the fine structure constant relates with the Kerr and Faraday rotation angle^24^,^25^.

Though our numerical calculations are based on TI/SiO_2_ multilayer structures, we have to emphasize that the mechanism presented here is very general. The TI/SiO_2_ multilayers may be substituted by magneto-optical materials^26^,^27^ or bi-anisotropic metamaterials^28^,^29^, where the gyrotropic permittivity (*g_z_*) and chirality (*C*) can play the role of *σ_xy_* here. For an example, we can show numerically that the polarization-conversion efficiency of MHA sandwiched between magneto-optical thin films with *g_z_* = 1 is almost equivalent to MHA sandwiched between two *N* = 20 TI/SiO_2_ multilayers (see supplementary material). We emphasize that the generalization of the mechanism to bi-anisotropic metamaterials should be important to extend the physics presented here to other frequency regime, since this kind of material has be realized in a much broader frequency range^30^.

To conclude, we demonstrate that a MHA sandwiched by two thin-layer materials characterized by general optical conductivities can serve as subwavelength quarter-wave and half-wave plates in Terahertz frequency regime. These are achieved by the hybrid resonances coupling the spoof surface plasmons with the currents in the thin layers.

## Methods

Following the geometry of the system, the tangential components of the electromagnetic fields in each region are given by Refs. 16, 17:

Region I:
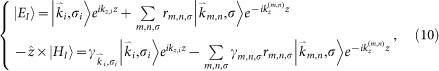
Region II:
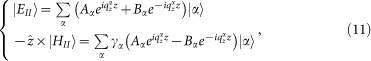
Region III:
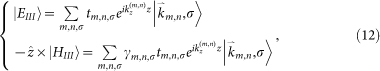
where region I and III are the open space, and region II is the MHA sandwiched between two thin layer materials. In the above, 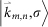 denotes the tangential components of Bloch waves in the free space, |*α*〉 denotes the tangential components of *α* waveguide modes in the holes, *Y_α_* is the admittance of the waveguide modes, *γ_m,n,σ_* is the admittance of the Bloch waves, *r_m,n,σ_* is the reflection coefficient of the Bloch mode labeled by m, n and σ, *t_m,n,σ_* is the transmission coefficient of the Bloch modes labeled by m, n and σ, *A_α_* is the coefficient for the *α* waveguide mode propagating forward, and *B_α_* is the coefficient for the *α* waveguide mode propagating backward.

The boundary conditions are as follows. The continuity condition for the electric field is given as:
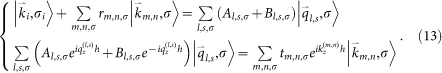
In the presence of the thin-layer materials, which support the in-layer currents, the boundary condition of magnetic fields at the interfaces of different regions would be different. To account the effect of in-layer currents, we firstly calculate these current and then put them into the boundary conditions. Following Ohm's law, the surface currents at the two interfaces are given by:
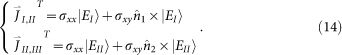
The boundary conditions of the magnetic fields at the two interfaces are then written as:
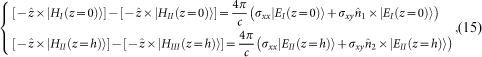
Combining Eqs. (10) and (12), after some simplifications, we arrive at Eq. (1) in the main text:
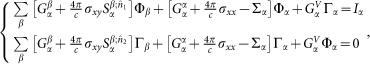
Where
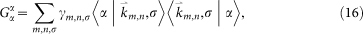



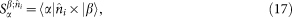



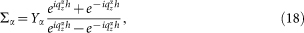



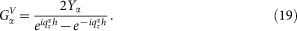


## Author Contributions

X.X. initializes the idea, X.X. and C.T.C. perform the theoretical analysis, H.M.L. and X.X. perform numerical calculations, and W.W. provides suggestions about experimental proposals. C.T.C. and W.W. supervise the project. X.X., C.T.C. and W.W. wrote the manuscript, and H.M.L. did proofreading.

## Supplementary Material

Supplementary Information Supplementary Information
