# Supplementary material for: Manipulation of the polarization of Terahertz wave in subwavelength regime
Source: Sci Rep. 2015 Feb 6;5:8306. doi: 10.1038/srep08306 (PMC4319153; doi:10.1038/srep08306)
Supplement: Supplementary Information [file srep08306-s1.pdf]

Supplementary material of "Manipulation of the polarization of Terahertz wave in subwavelength regime"

Xiao Xiao, Ho Ming Leung, C. T. Chan, and Weijia Wen

Department of Physics, Hong KongUniversity of Science and Technology
ClearWaterBay, Kowloon, Hong Kong, China

I. Derivations of Eq. (2) and Eq. (3) and relevant:

Equations (2) and (3) in the text are derived by taking into account only the fundamental waveguide modes in subwavelength hole. This approximation is quite good for subwavelength holes, because the fundamental waveguide modes decay slowest along the propagation direction. By taking such an approximation, Eq. (1) in the main text becomes:

$$\begin{cases} I_{0,1} = \Phi_{0,1}\left\{G_{0,1}^{0,1} + \dfrac{4\pi}{c}\sigma_{xx} - \Sigma_{0,1}\right\} + \Phi_{1,0}S_{0,1}^{1,0;\hat{n}_1} + G_{0,1}^{V}\Gamma_{0,1} \\ I_{1,0} = \Phi_{1,0}\left\{G_{1,0}^{1,0} + \dfrac{4\pi}{c}\sigma_{xx} - \Sigma_{0,1}\right\} + \Phi_{0,1}S_{1,0}^{0,1;\hat{n}_1} + G_{1,0}^{V}\Gamma_{1,0} \\ 0 = \Gamma_{0,1}\left\{G_{0,1}^{0,1} + \dfrac{4\pi}{c}\sigma_{xx} - \Sigma_{0,1}\right\} + \Gamma_{1,0}S_{0,1}^{1,0;\hat{n}_2} + G_{0,1}^{V}\Phi_{0,1} \\ 0 = \Gamma_{1,0}\left\{G_{1,0}^{1,0} + \dfrac{4\pi}{c}\sigma_{xx} - \Sigma_{0,1}\right\} + \Gamma_{0,1}S_{1,0}^{0,1;\hat{n}_2} + G_{1,0}^{V}\Phi_{1,0} \end{cases}. \quad (1)$$

where in the subscripts and superscript '0,1' indicates the transversal electric (TE) modes with indexes (0,1), and '1,0' presents the TE modes with indexes (1,0). The reflection and transmission coefficients relate to $\Phi_{0,1}$, $\Phi_{1,0}$, $\Gamma_{0,1}$ and $\Gamma_{1,0}$ as below: ($I_{0,1} = -I_{1,0}$)

$$\begin{cases} R_{xx} = \Phi_{0,1}I_{0,1} - 1 \\ R_{xy} = \Phi_{1,0}I_{1,0} \\ T_{xx} = \Gamma_{0,1}I_{0,1} \\ T_{xy} = \Gamma_{1,0}I_{1,0} \end{cases}. \quad (2)$$

Case I: quarter-wave plate

Then we assume $\hat{n}_1 = \hat{n}_2$ and solve Eq. (9) above to obtain the reflection and transmission coefficients:

$$\begin{cases} R_{xx} = \dfrac{I_{0,1}{}^2/2\left(G_S{}^3 + G_S G_C{}^2 - G_S G_V{}^2\right)}{G_S{}^4 + G_C{}^4 + G_V{}^4 + 2G_S{}^2 G_C{}^2 - 2G_S{}^2 G_V{}^2 + 2G_C{}^2 G_V{}^2} - 1 \\ R_{xy} = \dfrac{-I_{0,1}{}^2/2\left(G_C{}^3 + G_C G_S{}^2 + G_C G_V{}^2\right)}{G_S{}^4 + G_C{}^4 + G_V{}^4 + 2G_S{}^2 G_C{}^2 - 2G_S{}^2 G_V{}^2 + 2G_C{}^2 G_V{}^2} \\ T_{xx} = \dfrac{I_{0,1}{}^2/2\left(G_V{}^3 - G_V G_S{}^2 + G_V G_C{}^2\right)}{G_S{}^4 + G_C{}^4 + G_V{}^4 + 2G_S{}^2 G_C{}^2 - 2G_S{}^2 G_V{}^2 + 2G_C{}^2 G_V{}^2} \\ T_{xy} = \dfrac{I_{0,1}{}^2 G_V G_S G_C}{G_S{}^4 + G_C{}^4 + G_V{}^4 + 2G_S{}^2 G_C{}^2 - 2G_S{}^2 G_V{}^2 + 2G_C{}^2 G_V{}^2} \end{cases}.$$

where we define: $G_S = G_{0,1}^{0,1} + \dfrac{4\pi}{c}\sigma_{xx} - \Sigma_{0,1}$, $G_C = \dfrac{4\pi}{c}\sigma_{xy} S_{0,1}^{1,0;\hat{n}}$, $G_V = G_{0,1}^V$, and $I_{0,1}{}^2/2 = 2\left(8/9\pi^2\right)$. The above equations are Eq. (2) in the main text. Since the holes are subwavelength and we are interested in subwavelength regime, we notice that $\mathbf{Re}\,G_S \gg \mathbf{Im}\,G_S$, and $\mathbf{Im}\,G_S$ should be positive. On the other hand, according to Eq. (8d), $G_{0,1}^V$ is a pure imaginary number, and $\mathbf{Im}\,G_{0,1}^V$ is negative. Thus, the condition of $|T_{xx}| = |T_{xy}|$ can be approximated as:

$$-\left(\mathbf{Im}\,G_V\right)^2 + \left(\mathbf{Im}\,G_S\right)^2 + G_C{}^2 = \pm 2\left(\mathbf{Im}\,G_S\right)G_C,$$

which indicates that with the condition $G_c \pm \mathbf{Im}\,G_S = \pm\mathbf{Im}\,G_V$, the transmitted light is circularly polarized.

At the same time, the denominator of these coefficients can be approximated as:

$$\begin{aligned} & G_S{}^4 + G_C{}^4 + G_V{}^4 + 2G_S{}^2 G_C{}^2 - 2G_S{}^2 G_V{}^2 + 2G_C{}^2 G_V{}^2 \\ & \approx \left(\mathbf{Im}\,G_S\right)^4 + G_C{}^4 + \left(\mathbf{Im}\,G_V\right)^4 - 2\left(\mathbf{Im}\,G_S\right)^2 G_C{}^2 - 2\left(\mathbf{Im}\,G_S\right)^2\left(\mathbf{Im}\,G_V\right)^2 - 2\left(\mathbf{Im}\,G_V\right)^2 G_C{}^2 \\ & + i4\,\mathbf{Re}\,G_S\left[G_C{}^2\,\mathbf{Im}\,G_S + \left(\mathbf{Im}\,G_V\right)^2\mathbf{Im}\,G_S - \left(\mathbf{Im}\,G_S\right)^3\right] \\ & = \left[\left(\mathbf{Im}\,G_S\right) - \left(G_C + \left(\mathbf{Im}\,G_V\right)\right)\right]\left[\left(\mathbf{Im}\,G_S\right) + \left(G_C + \left(\mathbf{Im}\,G_V\right)\right)\right] \\ & \times\left[\left(\mathbf{Im}\,G_S\right) - \left(G_C - \left(\mathbf{Im}\,G_V\right)\right)\right]\left[\left(\mathbf{Im}\,G_S\right) + \left(G_C - \left(\mathbf{Im}\,G_V\right)\right)\right] \\ & + i4\,\mathbf{Re}\,G_S\left[G_C{}^2\,\mathbf{Im}\,G_S + \left(\mathbf{Im}\,G_V\right)^2\mathbf{Im}\,G_S - \left(\mathbf{Im}\,G_S\right)^3\right] \end{aligned}, \qquad (3)$$

which means that under the condition $G_c \pm \mathbf{Im} G_S = \pm \mathbf{Im} G_V$, the real part of the denominator simply becomes vanishing. Therefore, we arrive at the conclusion in the main text: the resonances of the system coincide with the polarization changes.

Case II: half-wave plate

By taking $\hat{n}_1 = -\hat{n}_2$, we solve Eq. (9) to obtain the reflection and transmission coefficients:

$$\begin{cases} R_{xx} = \dfrac{2\left(8/9\pi^2\right)G_S}{G_S{}^2 + G_C{}^2 - G_V{}^2} - 1 \\ R_{xy} = \dfrac{-2\left(8/9\pi^2\right)G_C}{G_S{}^2 + G_C{}^2 - G_V{}^2} \end{cases} \text{and} \begin{cases} T_{xx} = \dfrac{-2\left(8/9\pi^2\right)G_V}{G_S{}^2 + G_C{}^2 - G_V{}^2}, \\ T_{xy} = 0 \end{cases}$$

which is Eq. (5) in the main text. The expansion of the denominator to the order of $O(\mathbf{Re} G_S) = O\left(8/9\pi^2\right)$ gives:

$$G_S{}^2 + G_C{}^2 - G_V{}^2 \approx -\left|G_S\right|^2 + G_C{}^2 + \left|G_V\right|^2 + i2\left(8/9\pi^2\right)\mathbf{Im}\left(G_S\right).$$

At the resonance, we have $-\left|G_S\right|^2 + G_C{}^2 + \left|G_V\right|^2 = 0$. Thus, we end up with:

$$R_{xx} \approx \frac{i2\left(8/9\pi^2\right)\mathbf{Im}\left(G_S\right)}{i2\left(8/9\pi^2\right)\mathbf{Im}\left(G_S\right)} - 1 = 0.$$

Therefore, we analytically show that the polarization of the reflected light is rotated by 90° from the incident one, but the transmitted polarization keeps the same.

II. Analysis of strength and handedness of the transmitted and reflected lights for quarter-wave plate case:

The strength of the reflected and transmitted lights:

Following the expansion of the denominator in Eq. (11), we notice that at the resonant conditions the real part of denominator simply gets vanishing, and only the imaginary part is left. Thus, it is clear that R_{xx} is real, but R_{xy} is imaginary.

On the other hand, the condition for $\left|R_{xy}\right| = \left|T_{xy}\right|$ can be approximated as:

$$G_C{}^2-(\mathbf{Im}G_S)^2-(\mathbf{Im}G_V)^2=\pm(\mathbf{Im}G_V)(\mathbf{Im}G_S),$$

which means $G_c=\pm\left[(\mathbf{Im}G_S)\pm(\mathbf{Im}G_V)\right]$. This condition is equivalent to $G_c\pm\mathbf{Im}G_S=\pm\mathbf{Im}G_V$. Moreover, it also can be shown that $|R_{xx}+1|=|T_{xy}|$. Using the conservation condition, we have:

$$|T_{xx}|^2+|T_{xy}|^2+|R_{xx}|^2+|R_{xy}|^2=1\Rightarrow 4|T_{xy}|^2-2|T_{xy}|+1=1\Rightarrow|T_{xy}|=1/2.$$

This results means that at resonant frequencies: $|T_{xx}|=|T_{xy}|=|R_{xx}|=|R_{xy}|=\frac{1}{2}$. Thus, the reflected light is also circularly polarized! Moreover, under the resonant conditions $G_c\pm\mathbf{Im}G_S=\pm\mathbf{Im}G_V$, the strength of transmission light is equal to that of the reflected light.

The handedness of the reflected and transmitted lights:

To explore the handedness of the light, we explicitly calculate the transmission and reflection coefficients at resonant conditions. Since the denominators are the same, we will calculate the numerators of the coefficients. We notice that R_{xx} is a special coefficient for the following reasons: 1. R_{xx} is real; 2. $|R_{xx}|=\frac{1}{2}$; 3. $|R_{xx}+1|=|T_{xy}|=\frac{1}{2}$. These facts have been shown in the section "The strength of the reflected and transmitted lights". Thus, we can conclude that $R_{xx}\approx-\frac{1}{2}$ under all possible resonant conditions. We then calculate other coefficients for all possible resonant conditions.

(a) $(\mathbf{Im}G_S)-(G_C-(\mathbf{Im}G_V))=0\Rightarrow G_C=(\mathbf{Im}G_V)+(\mathbf{Im}G_S)$:

Then we take $G_C=(\mathbf{Im}G_V)+(\mathbf{Im}G_S)$ into R_{xx}:

$$
\begin{aligned}
R_{xx} &= \frac{I_{0,1}{}^{2}/2\left(G_S{}^{3}+G_S G_C{}^{2}-G_S G_V{}^{2}\right)}{D}-1 \\
&= \frac{I_{0,1}{}^{2}/2G_S\left[-\left(\mathbf{Im}\,G_S\right)^2+\left(\mathbf{Im}\,G_S+\mathbf{Im}\,G_V\right)^2+\left(\mathbf{Im}\,G_V\right)^2\right]}{D}-1 \\
&= \frac{I_{0,1}{}^{2}G_S\overbrace{\left(\mathbf{Im}\,G_V+\mathbf{Im}\,G_S\right)}^{G_C}\left(\mathbf{Im}\,G_V\right)}{D}-1 \\
&= \frac{I_{0,1}{}^{2}G_S G_C\left(\mathbf{Im}\,G_V\right)}{D}-1=\frac{I_{0,1}{}^{2}G_S G_C\left(G_V/i\right)}{D}-1=\frac{-iI_{0,1}{}^{2}G_S G_C G_V}{D}-1
\end{aligned}
\quad , \qquad (4)
$$

where we use the fact that at G_V is a pure imaginary number, so $\left(\mathbf{Im}\,G_V\right)=G_V/i$.

We notice that $T_{xy}=\frac{I_{0,1}{}^{2}G_V G_S G_C}{D}$. According to (12), we have $T_{xy}=\frac{I_{0,1}{}^{2}G_V G_S G_C}{D}=\frac{i}{2}$.

Then further taking $G_C=\left(\mathbf{Im}\,G_V\right)+\left(\mathbf{Im}\,G_S\right)$ into R_{xy} and T_{xx}, and we have:

$$
\begin{aligned}
R_{xy} &= \frac{-I_{0,1}{}^{2}/2\left(G_C{}^{3}+G_C G_S{}^{2}+G_C G_V{}^{2}\right)}{D} \\
&= \frac{-I_{0,1}{}^{2}/2G_C\left[\left(-\mathbf{Im}\,G_S-\mathbf{Im}\,G_V\right)^2-\left(\mathbf{Im}\,G_S\right)^2-\left(\mathbf{Im}\,G_V\right)^2\right]}{D} \\
&= \frac{-I_{0,1}{}^{2}G_C\,\mathbf{Im}\,G_S\,\mathbf{Im}\,G_V}{D} \\
&\approx \frac{I_{0,1}{}^{2}G_C G_S G_V}{D}=\frac{i}{2}
\end{aligned}
\quad ,
$$

where we use the fact that G_S can be regarded as a pure imaginary number in subwavelength regime. Similarly,

$$
\begin{aligned}
T_{xx} &= \frac{I_{0,1}{}^{2}/2\left(G_V{}^{3}-G_V G_S{}^{2}+G_V G_C{}^{2}\right)}{D} \\
&= \frac{I_{0,1}{}^{2}/2G_V\left[\left(-\mathbf{Im}\,G_V\right)^2+\left(\mathbf{Im}\,G_S\right)^2+\left(\mathbf{Im}\,G_V+\mathbf{Im}\,G_S\right)^2\right]}{D} \\
&= \frac{I_{0,1}{}^{2}G_V\,\mathbf{Im}\,G_S G_C}{D}=\frac{-iI_{0,1}{}^{2}G_V G_S G_C}{D}=\frac{1}{2}
\end{aligned}
\quad .
$$

Summarize what we have obtained: $T_{xx} = -R_{xx}$ and $T_{xy} = R_{xy}$. Considering that the opposite propagation direction of the transmitted and reflected lights, we find that they should be of the same handedness!

(b) $(\mathbf{Im}G_S) - (G_C + (\mathbf{Im}G_V)) = 0 \Rightarrow G_C = (\mathbf{Im}G_S) - (\mathbf{Im}G_V)$

The reflection coefficient R_{xx} is given by:

$$
\begin{aligned}
R_{xx} &= \frac{I_{0,1}{}^2/2\left(G_S{}^3 + G_S G_C{}^2 - G_S G_V{}^2\right)}{D} - 1 \\
&= \frac{I_{0,1}{}^2/2G_S\left[-(\mathbf{Im}G_S)^2 + (\mathbf{Im}G_S - \mathbf{Im}G_V)^2 + (\mathbf{Im}G_V)^2\right]}{D} - 1 \\
&= \frac{I_{0,1}{}^2 G_S(\mathbf{Im}G_V - \mathbf{Im}G_S)(\mathbf{Im}G_V)}{D} - 1 = \frac{-I_{0,1}{}^2 G_S G_C(\mathbf{Im}G_V)}{D} - 1 \\
&= \frac{iI_{0,1}{}^2 G_S G_C G_V}{D} - 1
\end{aligned}, \qquad (5)
$$

Thus, we have $T_{xy} = \frac{I_{0,1}{}^2 G_V G_S G_C}{D} = -\frac{i}{2}$.

For the other two coefficients:

$$
\begin{aligned}
R_{xy} &= \frac{-I_{0,1}{}^2/2\left(G_C{}^3 + G_C G_S{}^2 + G_C G_V{}^2\right)}{D} \\
&= \frac{-I_{0,1}{}^2/2G_C\left[(\mathbf{Im}G_S - \mathbf{Im}G_V)^2 - (\mathbf{Im}G_S)^2 - (\mathbf{Im}G_V)^2\right]}{D} \\
&= \frac{I_{0,1}{}^2 G_C\,\mathbf{Im}G_S\,\mathbf{Im}G_V}{D} \\
&= \frac{-I_{0,1}{}^2 G_C G_S G_V}{D} = \frac{i}{2}
\end{aligned},
$$

$$T_{xx} = \frac{I_{0,1}{}^2/2\left(G_V{}^3 - G_V G_S{}^2 + G_V G_C{}^2\right)}{D}$$
$$= \frac{I_{0,1}{}^2/2G_V\left[-\left(\mathbf{Im}\,G_V\right)^2 + \left(\mathbf{Im}\,G_S\right)^2 + \left(\mathbf{Im}\,G_S - \mathbf{Im}\,G_V\right)^2\right]}{D}$$
$$= \frac{I_{0,1}{}^2 G_V\,\mathbf{Im}\,G_S G_C}{D} = \frac{-iI_{0,1}{}^2 G_V G_S G_C}{D} = -\frac{1}{2}.$$

Thus, we have: $T_{xx} = R_{xx}$ and $T_{xy} = -R_{xy}$, which means at the resonance determined by $\left(\mathbf{Im}\,G_S\right) - \left(G_C + \left(\mathbf{Im}\,G_V\right)\right) = 0$ the handedness of transmitted light is the same with that of reflected light!

(c) $\left(\mathbf{Im}\,G_S\right) + \left(G_C + \left(\mathbf{Im}\,G_V\right)\right) = 0 \Rightarrow G_C = -\left(\mathbf{Im}\,G_S\right) - \left(\mathbf{Im}\,G_V\right)$

The reflection coefficient R_{xx} is given by:

$$\begin{aligned} R_{xx} &= \frac{I_{0,1}{}^2/2\left(G_S{}^3 + G_S G_C{}^2 - G_S G_V{}^2\right)}{D} - 1 \\ &= \frac{I_{0,1}{}^2/2G_S\left[-\left(\mathbf{Im}\,G_S\right)^2 + \left(-\mathbf{Im}\,G_S - \mathbf{Im}\,G_V\right)^2 + \left(\mathbf{Im}\,G_V\right)^2\right]}{D} - 1 \\ &= \frac{I_{0,1}{}^2 G_S\left(\mathbf{Im}\,G_V + \mathbf{Im}\,G_S\right)\left(\mathbf{Im}\,G_V\right)}{D} - 1 = \frac{-I_{0,1}{}^2 G_S G_C\left(\mathbf{Im}\,G_V\right)}{D} - 1 \\ &= \frac{iI_{0,1}{}^2 G_S G_C G_V}{D} - 1 \end{aligned}, \qquad (6)$$

From (14), we have $T_{xy} = \frac{I_{0,1}{}^2 G_V G_S G_C}{D} = -\frac{i}{2}$.

For the other two coefficients:

$$R_{xy} = \frac{-I_{0,1}{}^2/2\left(G_C{}^3 + G_C G_S{}^2 + G_C G_V{}^2\right)}{D}$$
$$= \frac{-I_{0,1}{}^2/2G_C\left[\left(-\mathbf{Im}\,G_S - \mathbf{Im}\,G_V\right)^2 - \left(\mathbf{Im}\,G_S\right)^2 - \left(\mathbf{Im}\,G_V\right)^2\right]}{D}$$
$$= \frac{-I_{0,1}{}^2 G_C\,\mathbf{Im}\,G_S\,\mathbf{Im}\,G_V}{D} = \frac{I_{0,1}{}^2 G_C G_S G_V}{D} = -\frac{i}{2},$$

$$T_{xx} = \frac{I_{0,1}{}^2 / 2\left(G_V{}^3 - G_V G_S{}^2 + G_V G_C{}^2\right)}{D}$$
$$= \frac{I_{0,1}{}^2 / 2G_V\left[-\left(\mathbf{Im}\,G_V\right)^2 + \left(\mathbf{Im}\,G_S\right)^2 + \left(-\mathbf{Im}\,G_S - \mathbf{Im}\,G_V\right)^2\right]}{D}$$
$$= \frac{-I_{0,1}{}^2 G_V\,\mathbf{Im}\,G_S G_C}{D} = \frac{iI_{0,1}{}^2 G_V G_S G_C}{D} = \frac{1}{2}.$$

To summarize this situation: $T_{xx} = -R_{xx}$ and $T_{xy} = R_{xy}$. Once again, we find that the handedness of transmitted light is the same with that of reflected light!

(d) $\left(\mathbf{Im}\,G_S\right) + \left(G_C - \left(\mathbf{Im}\,G_V\right)\right) = 0 \Rightarrow G_C = \left(\mathbf{Im}\,G_V\right) - \left(\mathbf{Im}\,G_S\right)$

The reflection coefficient R_{xx} is given by:

$$R_{xx} = \frac{I_{0,1}{}^2 / 2\left(G_S{}^3 + G_S G_C{}^2 - G_S G_V{}^2\right)}{D} - 1$$
$$= \frac{I_{0,1}{}^2 / 2G_S\left[-\left(\mathbf{Im}\,G_S\right)^2 + \left(-\mathbf{Im}\,G_S + \mathbf{Im}\,G_V\right)^2 + \left(\mathbf{Im}\,G_V\right)^2\right]}{D} - 1, \tag{7}$$
$$= \frac{I_{0,1}{}^2 G_S\left(\mathbf{Im}\,G_V - \mathbf{Im}\,G_S\right)\left(\mathbf{Im}\,G_V\right) = I_{0,1}{}^2 G_S G_C\left(\mathbf{Im}\,G_V\right)}{D} - 1$$
$$= \frac{-iI_{0,1}{}^2 G_S G_C G_V}{D} - 1$$

Thus, from (15) we have $T_{xy} = \dfrac{I_{0,1}{}^2 G_V G_S G_C}{D} = \dfrac{i}{2}$.

For the other two coefficients:

$$R_{xy} = \frac{-I_{0,1}{}^2 / 2\left(G_C{}^3 + G_C G_S{}^2 + G_C G_V{}^2\right)}{D}$$
$$= \frac{-I_{0,1}{}^2 / 2G_C\left[\left(-\mathbf{Im}\,G_S + \mathbf{Im}\,G_V\right)^2 - \left(\mathbf{Im}\,G_S\right)^2 - \left(\mathbf{Im}\,G_V\right)^2\right]}{D}$$
$$= \frac{I_{0,1}{}^2 G_C\,\mathbf{Im}\,G_S\,\mathbf{Im}\,G_V}{D} = \frac{-I_{0,1}{}^2 G_C G_S G_V}{D} = -\frac{i}{2},$$

$$T_{xx} = \frac{I_{0,1}{}^2/2\left(G_V{}^3 - G_V G_S{}^2 + G_V G_C{}^2\right)}{D}$$

$$= \frac{I_{0,1}{}^2/2G_V\left[-\left(\mathbf{Im}\,G_V\right)^2 + \left(\mathbf{Im}\,G_S\right)^2 + \left(-\mathbf{Im}\,G_S + \mathbf{Im}\,G_V\right)^2\right]}{D}$$

$$= \frac{-I_{0,1}{}^2 G_V\,\mathbf{Im}\,G_S G_C}{D} = \frac{iI_{0,1}{}^2 G_V G_S G_C}{D} = -\frac{1}{2}$$

.

Thus, we find: $T_{xx} = R_{xx}$ and $T_{xy} = -R_{xy}$. This indicates that under the condition $\left(\mathbf{Im}\,G_S\right) + \left(G_C - \left(\mathbf{Im}\,G_V\right)\right) = 0$, the handedness of the transmitted light is still the same with that of the reflected light!

Summarize all the 4 possible resonant conditions, and we have the conclusion that the handedness of the transmitted light keeps the same with that of the reflected light, which holds at all possible resonant conditions!

III. ***An example of MHA sandwiched between two magneto-optical thin films as a quarter wave plate:***

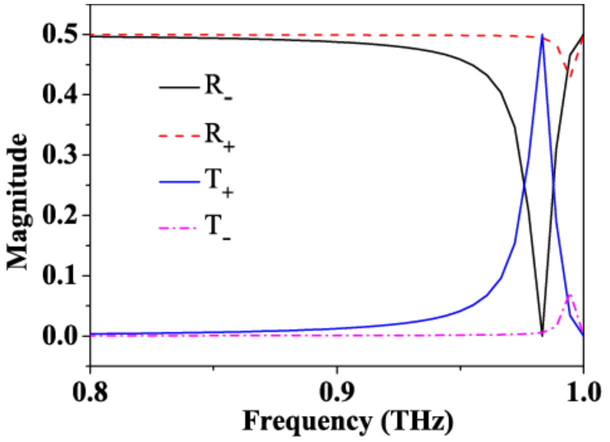

A1 the transmission and reflection of the left- and right-handed light though MHA sandwiched by two magneto-optical thin films.

To confirm the generality of the idea, we calculate the transmission and reflection from a MHA sandwiched by two magneto-optical thin films. We assume that the dielectric constant of the magneto-optical thin film is 3, and the gyrotropic permittivity is $\vec{g} = \left(g_x, g_y, g_z\right) = \left(0,0,1\right)$.

Then the electric field relates to the displacement field as $\vec{D} = \varepsilon\vec{E} + i\vec{E} \times \vec{g}$. We set the thickness of magneto-optical thin film is about 1/100 of that of MHA. Under this situation, the whole system is expected to be a quarter wave plate. The transmission and reflection from such a structure is calculated by a commercial finite element solver (***Comsol Multiphysics 4.2***) and shown in Fig.A1. One can see that at the resonant frequency both the reflected and transmitted waves are almost totally left-handed. The strength of the transmitted wave is equal to that of the reflected one. Moreover, by comparing the value of $P_{+/-}^{T}$, we find that the polarization conversion efficiency of the system is almost the same with MHA sandwiched between *N*=20 TI/SiO2 multilayers with $P_{+/-}^{T} \approx 0.99$.
